# Genome-wide single-nucleotide polymorphism data and mitochondrial hypervariable region 1 nucleotide sequence reveal the origin of the Akhal-Teke horse

**DOI:** 10.5713/ab.23.0044

**Published:** 2023-05-04

**Authors:** Zhoucairang Kang, Jinping Shi, Ting Liu, Yong Zhang, Quanwei Zhang, Zhe Liu, Jianfu Wang, Shuru Cheng

**Affiliations:** 1 College of Animal Science and Technology, Gansu Agricultural University, Lanzhou, 730070, China; 2 College of Veterinary Medicine, Gansu Agricultural University, Lanzhou 730070, China; 3 College of Life Science and Biotechnology, Gansu Agricultural University, Lanzhou 730070, China

**Keywords:** Akhal-Teke Horse, Central Asia, Middle East, Mitochondrial HVR-1, Single-nucleotide Polymorphism (SNP)

## Abstract

**Objective:**

The study investigated the origin of the Akhal-Teke horse using genome-wide single-nucleotide polymorphism (SNP) data and mitochondrial hypervariable region 1 (HVR-1) nucleotide sequences

**Methods:**

Genome-wide SNP data from 22 breeds (481 horses) and mitochondrial HVR-1 sequences from 24 breeds (544 sequences) worldwide to examine the origin of the Akhal-Teke horse. The data were analyzed using principal component analysis, linkage disequilibrium analysis, neighbor-joining dendrograms, and ancestry inference to determine the population relationships, ancestral source, genetic structure, and relationships with other varieties.

**Results:**

A close genetic relationship between the Akhal-Teke horse and horses from the Middle East was found. Analysis of mitochondrial HVR-1 sequences showed that there were no shared haplotypes between the Akhal-Teke and Tarpan horses, and the mitochondrial data indicated that the Akhal-Teke horse has not historically expanded its group. Ancestral inference suggested that Arabian and Caspian horses were the likely ancestors of the Akhal-Teke horse.

**Conclusion:**

The Akhal-Teke horse originated in the Middle East.

## INTRODUCTION

The Akhal-Teke horse is estimated to be at least 3,000 years old and is one of the oldest horse breeds in the world. Native to the southern region of Turkmenistan, the Akhal-Teke horse is adapted to harsh climatic conditions [1] and is famous for its beautiful appearance, familiarity with people, and shining coat color. The Akhal-Teke horse is one of the finest horse breeds in the world. Today, the Akhal-Teke horse population is slowly declining and is endangered. Currently, the Akhal-Teke is found mainly in Turkmenistan [2].

The Akhal-Teke is the best of a number of excellent horse breeds. The origin of this horse breed is highly controversial. Several hypotheses regarding the origin of the Akhal-Teke horse have been proposed based on archaeological and documentary evidence. The most plausible hypothesis is that the Akhal-Teke horse originated in Central Asia, in the area between the Caspian coast and the Fergana region [3]. It is thought that the earliest domestication of horses occurred in the Eurasian steppe around 3500 BCE, from where they spread across the Eurasian continent [4]. A study of DNA microsatellite genotyping of 2,024 Akhal-Teke horses from around the world showed that this horse breed has a high level of genetic diversity with abundant rare alleles at microsatellite DNA loci. Further analysis of the rare alleles showed that the Akhal-Teke horse is very similar to Tuvinia and Khakassia horses [5]. These results support the hypothesis of a Central Asian origin; studies investigating population changes associated with domestication from 273 ancient horse genomes also suggest that the western Eurasian steppe region is the home of the Akhal-Teke horse [6]. In contrast, there is a great deal of evidence indicating that the Akhal-Teke horse originated in the Middle East. The horse was widely documented in Middle Eastern epics and was referred to as the “supernatural horse,” “celestial horse,” or “bleeding stallion” [7]. Various molecular markers have been used in previous studies, which suggested that the Akhal-Teke horse belongs to the Middle Eastern horse breed clade [8–11]. Other archaeological studies at various sites suggest that the Akhal-Teke horse originated in Southwest Asia and migrated from Central Asia to the Middle East, or originated as a cross between the Tarpan and a domesticated native horse from Central Asia [12]. Thus, although several hypotheses have been proposed for the origin of the Akhal-Teke horse, many aspects surrounding its origin are unknown. In this study, we combined genotypic data from genome-wide high-density single-nucleotide polymorphism (SNP) arrays with equine mitochondrial hypervariable regions 1 (HVR-1) sequence data to elucidate the origin of the Akhal-Teke. The results provide new insights into the origin of the Akhal-Teke horse.

## MATERIALS AND METHODS

The experiment was approved by the Institutional Animal Care and Use Committee of the Gansu Agricultural University under permit number No.GAU-LC-2018-12.

### Datasets

The genome-wide SNP genotype data for Kazakh and Przewalskii horses were obtained from our previous study [13] that used the Illumina horse SNP 70 bead array (Illumina, San Diego, CA, USA) with the Illumina Equine SNP 50 bead array data (animalgenome.org) (Illumina, USA) from 20 Western horse breeds genotyped by Petersen et al [14,15]. The SNP70 and SNP50 arrays shared a total of 43,782 common markers and were used in subsequent analyses (Table 1). Quality control of the raw data was performed with PLINK v1.07 [16] and SNPs with call rates lower than 0.90, p<10^−5^ or MAF<0.01 were excluded. Finally, 36,734 sites remained after quality control.

### Population divergence

#### Linkage disequilibrium attenuation analysis

PopLDdecay software was used to measure the R^2^ of each population [17]. A curve of linkage disequilibrium (LD) change was drawn with R^2^ as the vertical axis and the physical distance between paired SNPs (<400 kb) as the horizontal axis.

#### Identity by descent (IBD) analyses

The frequency of shared haplotypes between Akhal-Teke horses and other horse breeds was calculated using IBDLD software (v3.37) [18]. The specific parameters were set as follows: “plinkbf int evolution -method GIBDLD -ploci 10 -nthreads 24 -step 0 -hiddenstates 3 -segment -length 10 -min 0.8”. The normalized identity by descent (nIBD) parameter was used for the statistics of haplotypes shared between Akhal-Teke horses and other horse breeds. The parameter Nibd = cIBD/tIBD was used in the formula, where cIBD represents all haplotypes shared by Akhal-Teke horses and other specific horse breeds, and tIBD is the number of pairs of Akhal-Teke and other specific horse breeds [19].

#### Formal test of ancestor admixture

The F4 ratio in the ADMIXTOOLS software package [20] was used to predict the most likely ancestral population. Default parameters were used in the analysis: the ratio f4 (A, O; X, C)/f4 (A, O; B, C) was calculated, where population X is the Akhal-Teke horse, A is the Exmoor horse with a relatively distant genetic relationship to X, O is the outgroup, the Przewalski horse, C is the thoroughbred, B is the other horse population The following is a list of the groups.

### Estimating the pedigree ratio of the Akhal-Teke horse

ALDER v1.03 [21] software was used to estimate the underlying proportions of genetic contributions from other horse breeds to the Akhal-Teke horse. This software determines the optimal ancestry in a reference population based on the difference in allele frequencies in the ancestral population and the correlation of SNPs in a weighted mixed target population. Here, Arabian and Caspian horses were used as the reference populations, and the Akhal-Teke horse as the target population. The parameters were mincount = 4, binsize = 0.0005, maxdis = 0.5, and fast_snpread = NO.

### Analysis of mitochondrial HVR-1 regions

The mitochondrial HVR-1 region sequences of 24 horse breeds from around the world were retrieved from the NCBI and manually edited and verified using DNASTAR software. Multiple alignments were performed using Mega7.0 to remove redundant sequences. The 24 horse breeds were divided into seven groups, namely, the Middle Eastern, Central Asian, European, Tarpan, Przewalski, and Akhal-Teke horse groups. The phylogenetic relationships were reconstructed using Popart software [22] and the differentiation index (F_ST_) between populations was calculated using Arlquine software, while mismatch analysis was performed for the Akhal-Teke horse to detect if there were expansion events in the history of the horse population.

## RESULTS

### Genetic relationships between the Akhal-Teke horse and other horse breeds of the world

**Figure 1:**
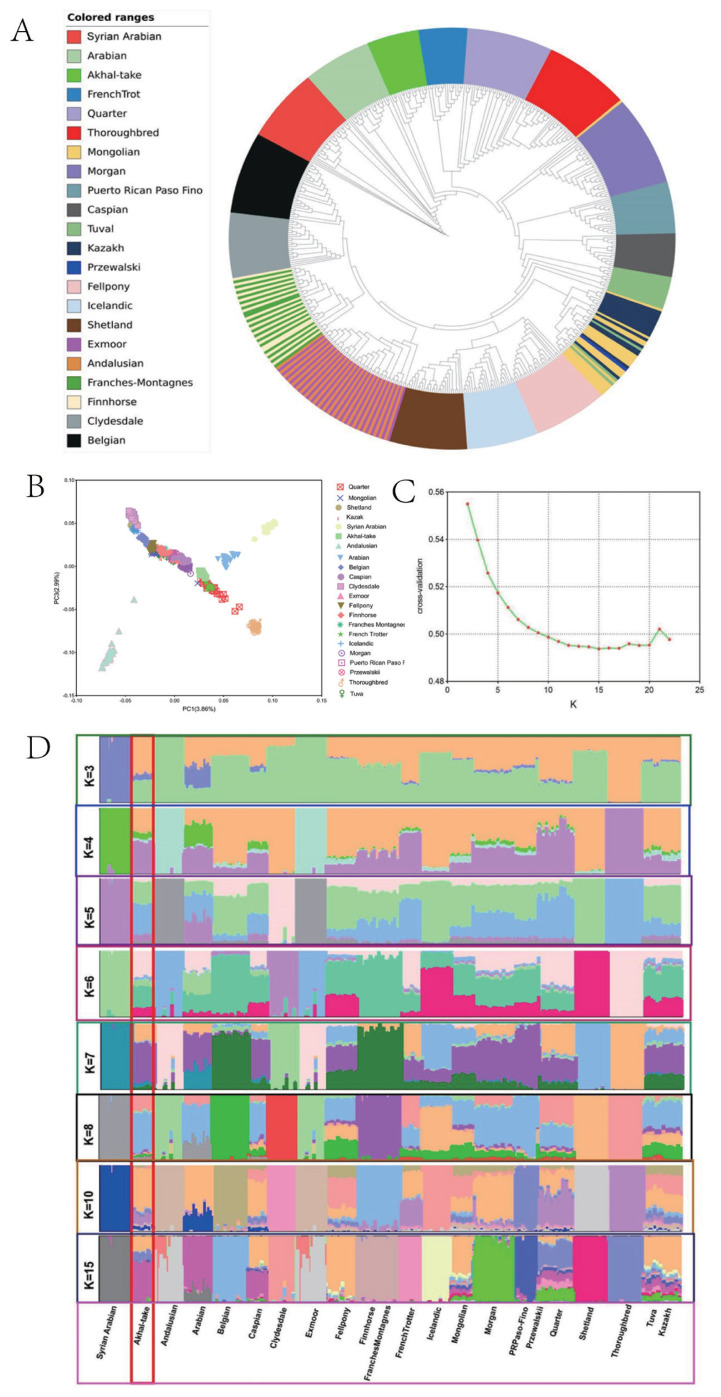
Genetic relationships between the Akhal-Teke horse and other horse breeds of the world. (A) Relationship between the Akhal-Teke horse and other horse populations shown by a neighbor-joining phylogenetic tree. Study populations are shown in different colors. (B) Principal component analysis of the Akhal-Teke horse and other horse populations. The different study populations are indicated by different colors. (C) Cross-validation under different K-values. (D) ADMIXTURE analysis of the Akhal-Teke horse and world horse populations. (K) Admixture plots of breeds analyzed with different numbers of assumed ancestors. Each column represents an individual. Columns represent individuals, groups of columns represent horse populations.

**Figure 2:**
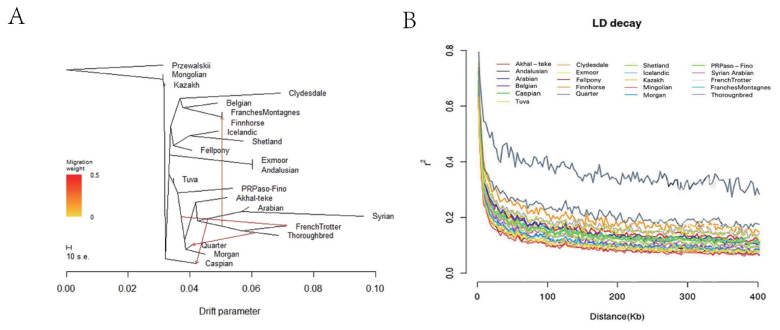
Population gene flow and the degree of domestication. (A) Migration showing four migration events in the Akhal-Teke horse and worldwide horse populations, shown by the TreeMix program; (B) linkage disequilibrium (LD) decay analysis of 22 horse populations from around the world, including Akhal-Teke horses. The different horse populations are represented by different colors.

The results of the nearest neighbor-joining tree showed that Akhal-Teke horses and Arabian horses form a cluster together, indicating that they are closely related. However, no close kinship was found between the Akhal-Teke horse and Caspian or Tuwa horses (Figure 1A), and principal component analysis results further confirmed the close genetic link between the Akhal-Teke horse and Middle Eastern horse populations (Figure 1B). Estimation of the genetic composition of the Akhal-Teke horse and other horses showed a minimum cross-validation statistic when K was set to 15. The results showed that the Akhal-Teke horse had a similar genetic background to Middle Eastern horse breeds, especially Arabian horses (Figure 1D). The Treemix results reflect several well-known gene flows (Figure 2A). For example, Thoroughbreds were observed to have had a significant genetic influence on Quarter horses, and Caspian horses were influenced by gene flow from Arabian horses. However, no gene exchange between Akhal-Teke and other horse breeds was detected and LD decay analysis showed that Akhal-Teke horses had LD decay levels similar to those of Tuva, Caspian, and Arabian horses. The results of this analysis indicated a moderate level of LD decay in the Akhal-Teke horses(Figure 2B).

### IBD analysis of the Akhal-Teke horse

**Figure 3:**
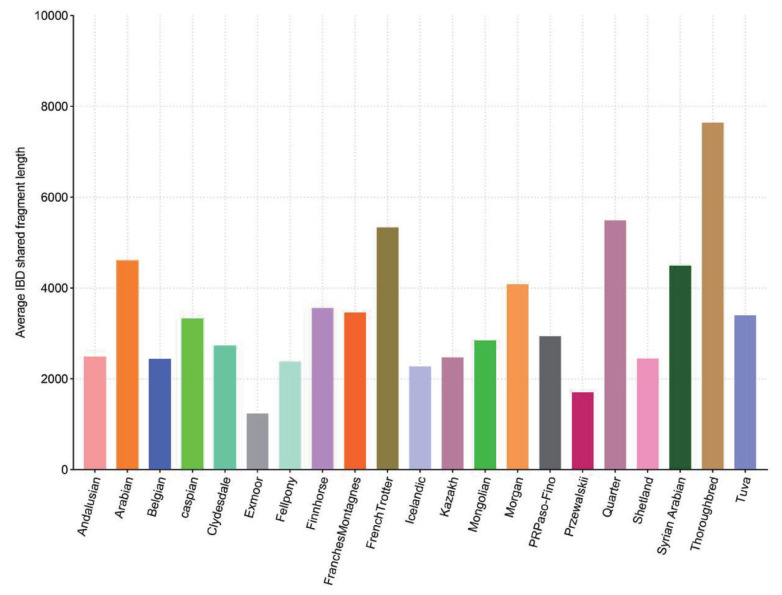
Estimated lengths of identity by descent (IBD) segments common to the Akhal-Teke and other horse breeds from around the world.

The results of the IBD shared-fragment analysis indicated the presence of relatively large shared fragments between the Akhal-Teke horse and Arabian horses and their derivatives (Thoroughbreds and Quarter horses). On the other hand, the length of shared fragments between the Tuva and Caspian horses and the Akhal-Teke horses was small. Nordic horses, especially the Exmoor breed, shared the fewest fragments with the Akhal-Teke horse (Figure 3).

### Estimation of possible ancestry

While the above analysis indicated a close genetic relationship between the Akhal-Teke horse and the Arabian horse and several other breeds, its ancestry was still undetermined. Therefore, the ADMIXTOOLS software package was applied to analyze the best ancestral composition. Exmoor and Thoroughbred horses were used as reference populations and the Akhal-Teke horse was used as the target population (X). The higher the output f4 ratio, the greater the likelihood of the reference population being ancestral to the target population. The results showed that Middle Eastern horses had a larger f4 ratio than the Central Asian horse population (Table 2).

### Evaluation of ancestry ratios of Akhal-Teke horses

**Figure 4:**
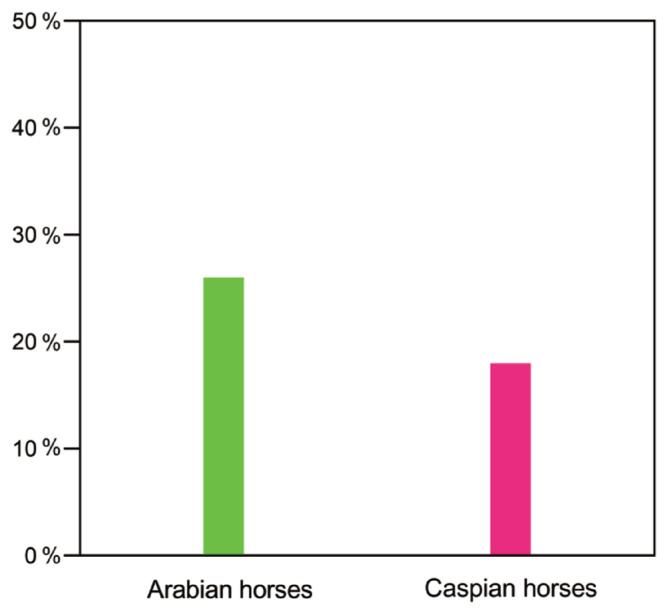
Evaluation of ancestry ratios of the Akhal-Teke horse. Arabian and Caspian horses were used as the reference populations, and the Akhal-Teke horse as the target population. Ancestry ratios are indicated by a percent sign.

The horse groups most likely to be ancestral to the Akhal-Teke horse were selected from the Middle Eastern and Central Asian horse populations. Alder v103 software was used to estimate the ancestral ratios of Arabian horses and Caspian horses in the Akhal-Teke horse. The Caspian and Arabian horses were used as the two reference groups, and the Akhal-Teke horse was used as the target group. The results showed that Arabian horses accounted for 26% of the ancestry of Akhal-Teke horses and Caspian horses accounted for 18% of the ancestry of Akhal-Teke horses (Figure 4).

### Mitochondrial HVR-1 regions

**Figure 5:**
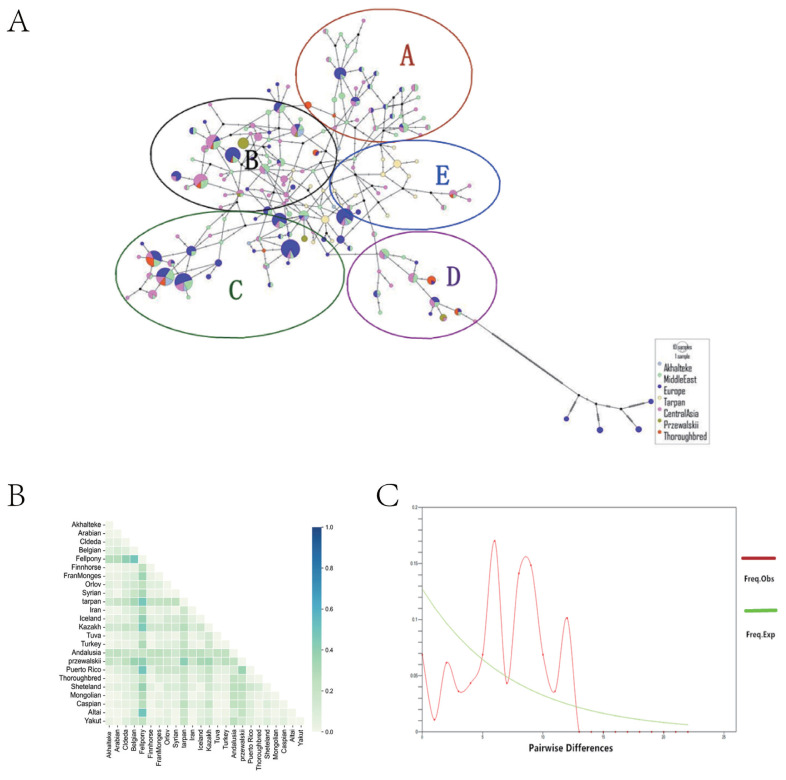
Results of the mitochondrial HVR-1 nucleotide sequence analysis. (A) Median-joining network between groups constructed in the mitochondrial HVR-1 region. Study populations are represented by different colors. All haplotypes were divided into five haplotype subgroups, with each subgroup circled and represented by a different letter. (B) Indicators of genetic differentiation between horse groups (F_ST_). Color intensity indicates the degree of differentiation between groups. (C) Mismatch analysis diagram of the Akhal-Teke horse. HVR-1, hypervariable region 1.

The 544 hypervariable regions of the 24 breeds were classified into seven major groups according to the geographical location of the population. These groups were the Middle Eastern, Akhal-Teke, Central Asian, European, Tarpan, Przewalski, and Thoroughbred horse groups. A total of 175 haplotypes were detected (Figure 5A). The results of the central join network showed that Akhal-Teke horses shared several haplotypes with horses from the Middle East and Central Asia. However, there were no common haplotypes between Akhal-Teke horses and Tarpan horses. The 175 haplotypes were also divided into five haplotype groups, A–E, with Tarpan horses found only in the E haplotype group and other horses marked for inclusion. The results of genetic differentiation between groups (F_ST_) analysis showed that the F_ST_ values between the Akhal-Teke and Tarpan, Przewalski, and Andalusian horses were relatively high compared to the other horse populations (Figure 5B). Mismatch analysis of the Akhal-Teke horse population showed multiple peaks (Figure 5C), indicating that there was no expansion in the history of this breed. Ultimately, the hypotheses that the Akhal-Teke horse is a cross between a local Central Asian breed and the Tarpan horse or that the Akhal-Teke horse migrated from Central Asia to the Middle East were not supported.

## DISCUSSION

The Akhal-Teke horse is one of the oldest and most famous horse breeds. It is now mainly distributed in Turkmenistan [2]. Most of the horses have golden chestnut hair, sometimes black or brown. The shoulder height is 150 to 163 cm. Akhal-Teke horses belong to the riding-horse category due to their sporting characteristics [23]. However, despite being one of the few purebred horse breeds in the world, the origin of the Akhal-Teke horse is largely unknown. The most likely hypothesis is that the Akhal-Teke horse originated in Central Asia, adjacent to the Eurasian steppe, where the earliest domestication of horses took place [24]. Conversely, the hypothesis that the Akhal-Teke originated in the Middle East is less plausible, given the paucity of archaeological evidence associated with Akhal-Teke horses found in the Middle East [12]. As the Turkmen horse [25] is the most important ancestor of the modern Akhal-Teke horse [26], another proposal is that the Akhal-Teke horse originated in Central Asia and was introduced to the Middle East. To further test this hypothesis, this study applied genome-wide SNP data from 22 breeds from around the world to analyze the genetic relationship between the Akhal-Teke horse and other breeds. The results suggested that the Akhal-Teke horse is not closely related to the Caspian or Tuva horse and that there was little genetic exchange between the two, which does not support the longstanding hypothesis that the Akhal-Teke horse originated in Central Asia [27]. However, closer genetic links between the Akhal-Teke horse and horses from the Middle East were found. Genetic exchanges between the Akhal-Teke horse and other horse breeds could not be detected by the Treemix analysis, which may be due to the relatively limited geographic distribution of the Akhal-Teke horse. Shared IBD fragment analysis of the Akhal-Teke horse provided further evidence that Middle Eastern horses are more likely to be the ancestors of the Akhal-Teke horse than other horses with the admixture results indicating that both Caspian and Arabian horses have similar genetic backgrounds to the Akhal-Teke horse. Furthermore, the ancestry estimates showed that Arabian horses contributed a greater proportion to the ancestry of the Akhal-Teke horse. The geographical distributions of the Arabian and Akhal-Teke horses almost overlap, which would have facilitated genetic exchange between the two breeds. These results are consistent with fossil finds reported in the literature that the Libyan peninsula, adjacent to the Middle East, played an essential role in the process of horse domestication as a major center of domestication [24]. The fact that the modern Akhal-Teke breed shares haplogroups with horses from Turkmenistan and Kazakhstan is more clearly delineated by the mitochondrial data, which also points to the likelihood that the modern Middle East is the likely origin of the Akhal-Teke horse [2,10].

Due to the lack of whole-genome SNP array data for the Tarpan horse, data on the mitochondrial HVR-1 region of the Tarpan horse [16,28–30] was obtained from NCBI. The genetic relationship between the Akhal-Teke and Tarpan horses was examined. The haplotypes were divided into five groups. All Tarpan horses were found in the E haplotype group, and no haplotypes were shared between Tarpan horses and other horses [29]. Therefore, our mtDNA results do not support the hypothesis that the Akhal-Teke horse is a hybrid between the Tarpan horse and a local Central Asian horse. This result is also consistent with the results from the genome-wide SNP data analysis in this study. Furthermore, the mtDNA data also showed that there was no historical group expansion of the Akhal-Teke horse, which did not support the hypothesis that the Akhal-Teke migrated from Central Asia to the Middle East. Thus, the results from both the genomic and mtDNA data suggest that the Akhal-Teke most likely originated in the Middle East.

## CONCLUSION

In this study, data from horse whole-genome SNP array chips and mitochondrial hypervariable regions indicated that the Akhal-Teke horse may have originated in the Middle East rather than in Central Asia. The study also found no support for the hypothesis that the breed was derived from a historical migration from Central Asia to the Middle East, or that it originated from a hybrid between the Tarpan horse and a domesticated local horse breed from Central Asia.

## Figures and Tables

**Table 1:** Information on the horse breeds included in this study

Breed	Population size	Geographic origin	Data source
Syrian Arabian	27	Syrian	Almarzook et al [15]
Akhal-Teke	18	Turkmenistan	Petersen et al [14]
Andalusian	24	Spain	Petersen et al [14]
Arabian	24	Middle East	Petersen et al [14]
Belgian	30	Belgium	Petersen et al [14]
caspian	16	Persia	Petersen et al [14]
Clydesdale	24	Scotland	Petersen et al [14]
Exmoor	24	England	Petersen et al [14]
Fellpony	26	England	Petersen et al [14]
Finnhorse	18	Finland	Petersen et al [14]
Franches-Montagnes	18	Switzerland	Petersen et al [14]
FrenchTrotter	17	France	Petersen et al [14]
Icelandic	25	Iceland	Petersen et al [14]
Mongolian	19	Mongolian	Petersen et al [14]
Morgan	33	United States	Petersen et al [14]
Puerto RicanPaso Fino	19	Puerto Rico	Petersen et al [14]
Przewalskii	1	China	Petersen et al [14]
Quarter	30	United States	Petersen et al [14]
Shetland	27	Scotland	Petersen et al [14]
Thoroughbred	30	United States	Petersen et al [14]
Tuva	15	Siberia	Petersen et al [14]
Kazakh	16	Middle East	Ma et al [13]

**Table 2:** Analysis of the F4 ratio of Akhal-Teke horses

A	O	X	C	A	O	B	C	F4 ratio	std.err	Z (null = 0)
Exmoor	Przewalskii	Akhal-teke	Thoroughbred	Exmoor	Przewalskii	Shetland	Thoroughbred	0.368	0.085	4.345
Exmoor	Przewalskii	Akhal-teke	Thoroughbred	Exmoor	Przewalskii	Syrian Arabian	Thoroughbred	0.511	0.170	3.000
Exmoor	Przewalskii	Akhal-teke	Thoroughbred	Exmoor	Przewalskii	Arabian	Thoroughbred	0.951	0.279	3.413
Exmoor	Przewalskii	Akhal-teke	Thoroughbred	Exmoor	Przewalskii	Tuva	Thoroughbred	0.376	0.080	4.676
Exmoor	Przewalskii	Akhal-teke	Thoroughbred	Exmoor	Przewalskii	Andalusian	Thoroughbred	−0.139	0.028	−5.054
Exmoor	Przewalskii	Akhal-teke	Thoroughbred	Exmoor	Przewalskii	caspian	Thoroughbred	0.485	0.104	4.661
Exmoor	Przewalskii	Akhal-teke	Thoroughbred	Exmoor	Przewalskii	Mongolian	Thoroughbred	0.262	0.068	3.851
Exmoor	Przewalskii	Akhal-teke	Thoroughbred	Exmoor	Przewalskii	Kakzh	Thoroughbred	0.291	0.072	4.033
Exmoor	Przewalskii	Akhal-teke	Thoroughbred	Exmoor	Przewalskii	Icelandic	Thoroughbred	0.349	0.085	4.123
Exmoor	Przewalskii	Akhal-teke	Thoroughbred	Exmoor	Przewalskii	Quarter	Thoroughbred	1.249	0.251	4.968
Exmoor	Przewalskii	Akhal-teke	Thoroughbred	Exmoor	Przewalskii	FrenchTrotter	Thoroughbred	1.042	0.344	3.034
Exmoor	Przewalskii	Akhal-teke	Thoroughbred	Exmoor	Przewalskii	Belgian	Thoroughbred	0.473	0.100	4.713
Exmoor	Przewalskii	Akhal-teke	Thoroughbred	Exmoor	Przewalskii	Clydesdale	Thoroughbred	0.714	0.203	3.515
Exmoor	Przewalskii	Akhal-teke	Thoroughbred	Exmoor	Przewalskii	Fellpony	Thoroughbred	0.371	0.082	4.518
Exmoor	Przewalskii	Akhal-teke	Thoroughbred	Exmoor	Przewalskii	Finnhorse	Thoroughbred	0.662	0.127	5.219
Exmoor	Przewalskii	Akhal-teke	Thoroughbred	Exmoor	Przewalskii	FranchesMontagnes	Thoroughbred	0.649	0.123	5.260
Exmoor	Przewalskii	Akhal-teke	Thoroughbred	Exmoor	Przewalskii	Morgan	Thoroughbred	0.569	0.120	4.739
Exmoor	Przewalskii	Akhal-teke	Thoroughbred	Exmoor	Przewalskii	PRPaso-Fino	Thoroughbred	0.533	0.113	4.728

## Data Availability

The access numbers of 544 mitochondrial HVR-1 sequences of 24 breads from NCBI are summarized in the excel table. Mitochondrial HVR-1 Nucleotide Sequence.xlsx
